# Improvement of Rice Biomass Yield through QTL-Based Selection

**DOI:** 10.1371/journal.pone.0151830

**Published:** 2016-03-17

**Authors:** Kazuki Matsubara, Eiji Yamamoto, Nobuya Kobayashi, Takuro Ishii, Junichi Tanaka, Hiroshi Tsunematsu, Satoshi Yoshinaga, Osamu Matsumura, Jun-ichi Yonemaru, Ritsuko Mizobuchi, Toshio Yamamoto, Hiroshi Kato, Masahiro Yano

**Affiliations:** 1 NARO Institute of Crop Science, Tsukuba, Ibaraki 305–8518, Japan; 2 National Institute of Agrobiological Sciences, Tsukuba, Ibaraki 305–8602, Japan; 3 NARO Agricultural Research Center, Niigata 943–0154, Japan; Aberystwyth University, UNITED KINGDOM

## Abstract

Biomass yield of rice (*Oryza sativa* L.) is an important breeding target, yet it is not easy to improve because the trait is complex and phenotyping is laborious. Using progeny derived from a cross between two high-yielding Japanese cultivars, we evaluated whether quantitative trait locus (QTL)-based selection can improve biomass yield. As a measure of biomass yield, we used plant weight (aboveground parts only), which included grain weight and stem and leaf weight. We measured these and related traits in recombinant inbred lines. Phenotypic values for these traits showed a continuous distribution with transgressive segregation, suggesting that selection can affect plant weight in the progeny. Four significant QTLs were mapped for plant weight, three for grain weight, and five for stem and leaf weight (at α = 0.05); some of them overlapped. Multiple regression analysis showed that about 43% of the phenotypic variance of plant weight was significantly explained (*P* < 0.0001) by six of the QTLs. From F_2_ plants derived from the same parental cross as the recombinant inbred lines, we divergently selected lines that carried alleles with positive or negative additive effects at these QTLs, and performed successive selfing. In the resulting F_6_ lines and parents, plant weight significantly differed among the genotypes (at α = 0.05). These results demonstrate that QTL-based selection is effective in improving rice biomass yield.

## Introduction

By 2050, we will need to feed two billion more people than at present [1, 2]. How can we accomplish that task? Rice (*Oryza sativa* L.) is the staple food for more than half of the world’s population, particularly in Asia (Ricepedia, http://ricepedia.org/rice-as-food/the-global-staple-rice-consumers). In some Asian countries such as South Korea and Japan, rice is used for multiple purposes, including flour, livestock feed (including whole-crop silage), biofuel, and conservation of paddy fields. For these reasons, further improvement of rice yield potential, including biomass, is a major challenge for breeders and geneticists. To our knowledge, however, little is known about the genetic architecture of rice biomass yield, except for the *semi-dwarfing 1* gene [3, 4]. Similar to animal quantitative traits [5], rice biomass yield (including grain yield) is governed by quantitative trait loci (QTLs) with small additive effects and greatly varies among individuals in variable and nonuniform environments. For yield and other traits under this type of genetic control (many QTLs with small effects), phenotypic values are continuously distributed in segregating populations and do not show simple Mendelian inheritance [6, 7].

More than 20 years ago, it was suggested that marker-assisted selection (MAS) would allow integration of molecular genetics and conventional phenotype-based selection [8]. Indeed, MAS is now widely used in rice breeding for improvement of traits, such as disease and insect resistance, for which the phenotypes can be associated with the genotypes of DNA markers [9, 10]. Recent advances in rice genomics are improving our understanding of the evolution and function of the rice genome and are facilitating rice improvement [11–14]; maximizing the use of such genomic data is necessary to continue increasing rice biomass yield.

Using single-nucleotide polymorphisms detected by high-throughput resequencing, we are currently assessing the effectiveness of genomics-assisted selection approaches such as QTL analysis, association study, genomic estimation of breeding value, and haplotype analysis based on pedigree, in the improvement of rice biomass yield [15, 16]. In the present study, we first analyzed QTLs for biomass yield and related traits using recombinant inbred lines (RILs) derived from a cross between the high-yielding cultivars ‘Tachisugata’ (TS) and ‘Hokuriku 193’ (H193), which are used mainly for livestock feeding. Both cultivars were produced by inter-subspecific crosses between *O*. *sativa* ssp. *japonica* and *indica* cultivars and not only produce high grain yield but also have large stems and leaves [16–18]. Using a large F_2_ population, we then divergently selected lines that carried alleles with positive or negative additive effects at several of the detected QTLs. Finally, we compared the biomass yield of the selected lines to evaluate the effectiveness of QTL-based selection in improving biomass yield.

## Materials and Methods

### Plant materials and experimental design

**Fig 1:**
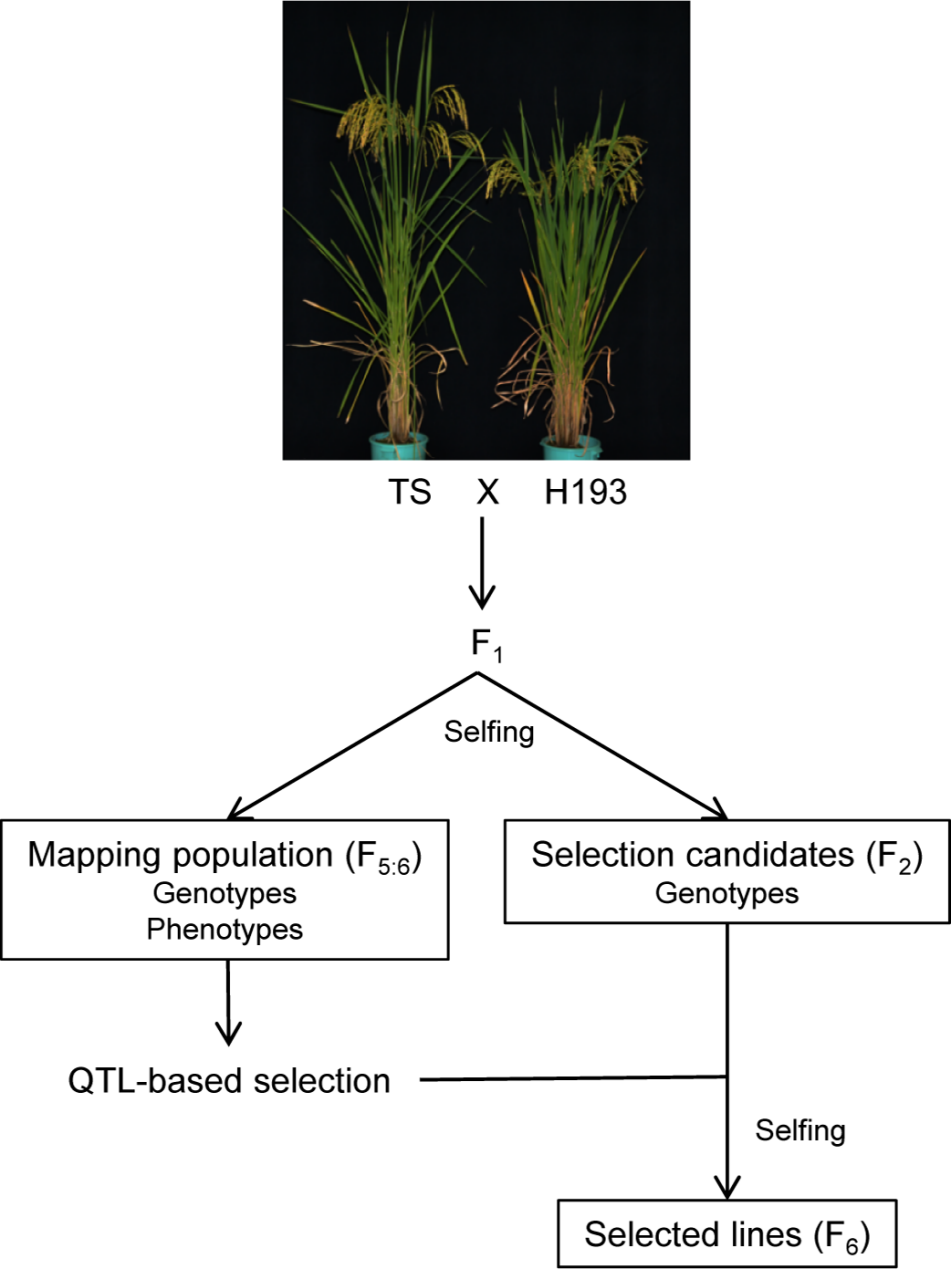
Diagram of QTL-based selection. Representative plants of the parental cultivars (~30 days after heading) are shown. TS, ‘Tachisugata’; H193, ‘Hokuriku 193’.

The experimental design is depicted in Fig 1. Self-progeny derived from a cross between TS and H193 were used. For QTL mapping, a set of F_5_ individuals (*N* = 191) was genotyped and then selfed to produce RILs. The trait value of a genotyped individual was estimated as the mean value of the resulting F_6_ family (*i*.*e*., F_5:6_ design). For phenotypic evaluation, RILs, parents, and their F_1_ progeny were grown in two replications (three rows of 14 individuals each *per* plot) in summer in a paddy field (Tsukubamirai, Japan). Seeds were sown in late April and 30-day-old seedlings were transplanted in the field with a spacing of 15 cm between plants within each row and 30 cm between rows. To avoid border effects, only the middle 10 individuals in the central row of each plot were analyzed.

To evaluate the effect of QTL-based selection on biomass yield, we raised more F_2_ individuals (*N* = 468) of the same TS × H193 cross and selected lines that carried alleles with positive or negative additive effects at detected QTLs (Fig 1). Selected lines were selfed by single-seed descent up to the F_6_ generation (Fig 1). Each plot had four rows (14 individuals *per* row), and only the middle individuals (10 *per* row) in the central two rows of each plot were analyzed.

### Genotyping

Genomic DNA was extracted from 2-month-old seedlings by the cetyltrimethylammonium bromide method [19]. From a previously published single nucleotide polymorphism (SNP) data set [16, 20]; Q-TARO database, http://qtaro.abr.affrc.go.jp/, a subset of 192 SNPs polymorphic between TS and H193 was selected and used for genotyping. In this subset, 175 SNPs were available for QTL analysis (S1 File). For QTL-based selection, a subset of 384 SNPs was used for genotyping, of which 344 SNPs were available in the F_2_ population [21]. In the F_6_ generation, QTL genotypes of the selected F_2_-derived lines were confirmed by determining the genotypes of the nearest SNP markers by direct sequencing of PCR products (S1 Table). Genotyping was performed on an Illumina GoldenGate BeadArray platform (Illumina Inc., San Diego, CA, USA).

### Phenotyping

Three biomass traits were evaluated: plant weight (aboveground parts only, PW, g), grain weight (GW, g), and stem and leaf weight (SLW, g). For each trait, 10 mature plants were bulked, dried for 48 h at 80°C, and weighed.

The following 11 morphological and physiological traits were also evaluated: harvest index (HI, %), culm length (CL, cm), panicle length (PL, cm), flag leaf length (FLL, cm), panicle number (PN), spikelet number per panicle (SN), 1000-grain weight (1000GW, g), spikelet fertility (SF, %), chlorophyll content (SPAD value), days to heading (DTH), and nonstructural carbohydrate content (NSC, %). HI was calculated as GW/PW × 100. For CL, PL, FLL, PN, SN, SF, and SPAD, phenotypic values of 10 plants per RIL were used to calculate means. For 1000GW, first, 100-grain weight was measured with four replications and then the mean was transformed to 1000-grain weight. DTH was scored as days from germination to heading (when 5 of the 10 plants had headed). For QTL and other analyses, means of two replications for each trait were used. NSC was measured according to [22]. A five-plant bulk (from another one of the three rows described above) was harvested at the yellow-ripening stage (approximately 30 days after heading) and was used to determine NSC.

### QTL analysis

A linkage map was constructed using RILs (*N* = 191). Linkage order and genetic distances of 175 marker loci were calculated with MAPMAKER/Exp 3.0 [23]. Residual heterozygotes were considered as missing data.

QTL analyses were performed using composite interval mapping as implemented in WinQTL Cartographer version 2.5 [24] with a significance level of α = 0.05. QTLs were added using forward and backward regression with the standard model (model 6) for up to five control markers. A window size of 10 cM was used with a walk speed of 2 cM. Significant LOD scores were assigned for each trait following permutation tests with 1000 replicates [25]. Box–Cox (for GW, HI, CL, FLL, 1000GW, and DTH) or arcsine (for SF) transformations were conducted for phenotypic data where normality was rejected by the Shapiro–Wilk test (S1 Fig) [26].

### Statistical analysis

Phenotypic correlations among the biomass-related traits were evaluated using Pearson’s correlation. The significance of each correlation was determined using *t* test with control of the false discovery rate for multiple tests [27]. Principal component analysis on the correlation matrix of line means for the 11 traits were also conducted.

To determine the total variation of PW explained by the significant QTLs for PW, GW, and SLW, multiple regression analysis of the first two principal components (PC1 and PC2) was performed. The minimum corrected Akaike information criterion was used to choose the best model. Path diagrams were generated according to Sokal and Rohlf [26].

To test whether QTL × QTL interaction was involved in the phenotypic variation of each trait in RILs, we performed two-way analysis of variance for all traits and PCs, using the genotypes of the SNPs nearest to the detected QTLs. Significance levels were corrected on the basis of the false discovery rate (α = 0.05) for multiple testing according to the number of interaction tests [27]. Significance of the difference for each trait value between genotypes was determined by the Tukey–Kramer HSD test.

All statistical analyses were performed using JMP 9 software (SAS Institute Inc., Cary, NC, USA

## Results

### Phenotypic variations of traits

Phenotypic variation of 14 traits in the parents, their F_1_ progeny, and RILs are summarized in Table 1. For PW, GW, and SLW (traits directly associated with biomass yield), there were no significant differences between the means of the parents. However, F_1_ plants showed larger values of these traits than their parents, suggesting that at least some QTLs underlying these traits are different between the parents. In contrast, the differences in CL, PL, PN, SN, 1000GW, SF, DTH, and NSC between the parents were significant. Transgressive segregation was evident in RILs for all evaluated traits, supporting the hypothesis that different alleles at the QTLs contribute to the traits in the two parents (Table 1, S1 Fig).

**Table 1:** Phenotypic variation of traits.

Trait	Abbreviation	Parent (*n* = 9)		Significance	F1 (*n* = 2)	RILs (*N* = 191)
		Tachisugata	Hokuriku 193			
		Mean (95% C.I.)	Mean (95% C.I.)		Mean (two trait values)	Mean (min.–max.)
Plant weight (g)	PW	826.9 (792.9–861.0)	861.9 (822.3–901.5)	*n*.*s*.	921.5 (889.0/954.0)	830.5 (544.4–1137.0)
Grain weight (g)	GW	330.7 (313.1–348.2)	348.2 (325.3–371.1)	*n*.*s*.	404.5 (383.0/426.0)	293.8 (113.0–405.0)
Stem and leaf weight (g)	SLW	496.3 (478.2–514.3)	513.7 (492.5–534.9)	*n*.*s*.	517.0 (506.0/528.0)	536.8 (382.0–760.5)
Harvest index (%)	HI	40.0 (39.2–40.7)	40.3 (39.1–41.5)	*n*.*s*.	43.9 (43.1/44.7)	35.4 (16.4–48.2)
Culm length (cm)	CL	105.1 (103.2–107.0)	90.6 (89.4–91.8)	1.14E-10	119.4 (119.6/119.2)	102.0 (64.5–136.6)
Panicle length (cm)	PL	27.0 (26.5–27.5)	28.3 (27.9–28.8)	1.69E-04	29.7 (30.1/29.2)	28.4 (22.8–36.6)
Flag leaf length (cm)	FLL	36.2 (34.0–38.3)	34.8 (33.9–35.8)	*n*.*s*.	37.9 (37.8/37.9)	37.4 (29.3–45.9)
Panicle number (count)	PN	8.3 (7.9–8.6)	9.3 (9.1–9.4)	3.61E-05	8.1 (8.0/8.2)	8.6 (5.8–11.9)
Spikelet number per panicle (count)	SN	216.1 (208.1–224.1)	193.7 (185.2–202.2)	4.34E-04	242.3 (250.5/234.1)	198.3 (116.3–320.4)
1000-grain weight (g)	1000GW	32.5 (32.2–32.8)	28.7 (28.6–28.9)	2.12E-13	32.2 (32.2/32.3)	29.0 (22.6–36.3)
Spikelet fertility (%)	SF	81.4 (79.1–83.6)	85.5 (82.6–88.5)	2.05E-02	88.2 (85.7/90.7)	80.3 (49.2–94.2)
Chlorophyll content (SPAD value)	SPAD	41.0 (40.2–41.8)	40.3 (39.5–41.1)	*n*.*s*.	43.5 (43.2/43.9)	41.3 (33.2–47.1)
Days to heading (days)	DTH	109.1 (108.5–109.7)	110.6 (110.1–111.0)	4.09E-04	108.0 (108.0/108.0)	110.6 (103.5–122.0)
Non–structural carbohydrate content (%)	NSC	28.5 (25.8–31.3)	33.7 (30.3–37.0)	1.19E-02	31.1 (29.3/33.0)	36.3 (17.9–53.6)

Averages of bulk consisting of 10 plants were shown for PW, GW and SLW. Differences between means ('Tachisugata' vs 'Hokuriku 193') were compared by the two-tailed Student's *t-test*.

### Phenotypic relationships between traits

**Fig 2:**
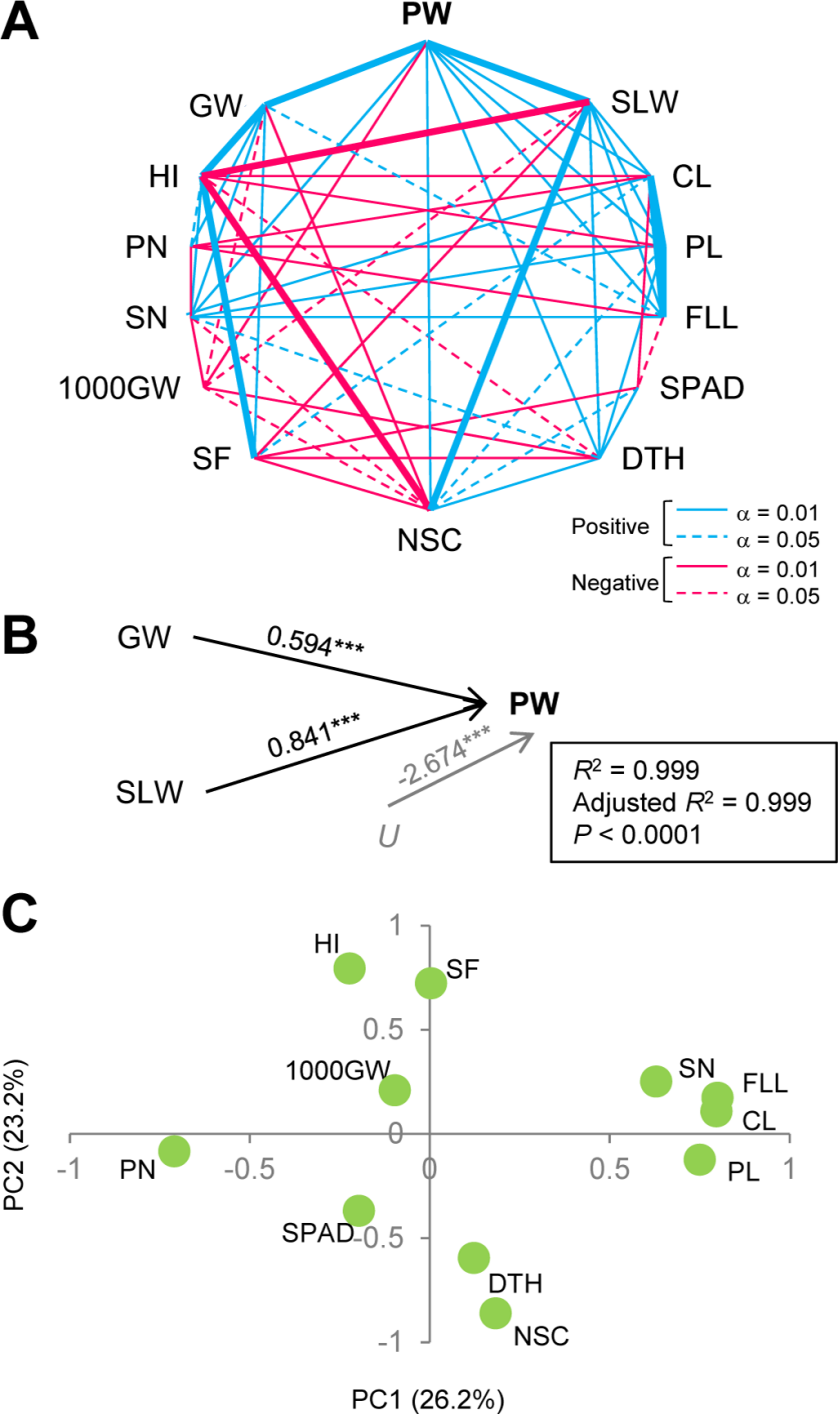
Phenotypic correlations between biomass-related traits quantified in recombinant inbred lines. (A) Relationships between the traits according to Pearson’s correlation coefficients. Two traits that showed significant correlation are connected to each other. Significant correlation coefficients are indicated by solid lines (more than ±0.212; α = 0.01) or broken lines (more than ±0.156; α = 0.05). Correlation coefficients of more than ±0.500 are shown by thick solid lines. PW, plant weight; SLW, stem and leaf weight; CL, culm length; PL, panicle length; FLL, flag leaf length; SPAD, chlorophyll content; DTH, days to heading; NSC, non-structural carbohydrate content; SF, spikelet fertility; 1000GW, 1000-grain weight; SN, spikelet number per panicle; PN, panicle number; HI, harvest index; GW, grain weight. (B) Path diagram showing two independent variables, GW and SLW, and the residual variable *U*, affecting plant weight (PW). Numbers indicate partial regression coefficients. ***, *P* < 0.0001. (C) Biplot based on principal component analysis for the traits. The percent variation explained by each principal component is shown in parentheses.

To investigate the relationships between the 14 traits, Pearson’s correlation coefficients between them were calculated for RILs (Fig 2A, S2 Table). All traits except SPAD were significantly correlated with at least one biomass trait (PW, GW, or SLW). Positive correlations between CL, PL, and FLL were marked (*r* > 0.5). SPAD was positively correlated with DTH and NSC and negatively correlated with CL, FLL, and SF.

It was not unexpected that significant correlations between PW and GW (*r* = 0.546, *P* < 0.0001) and between PW and SLW (*r* = 0.805, *P* < 0.0001) were observed, because PW is the sum of GW and SLW. Of note is the closer relationship between PW and SLW than between PW and GW (S2 Table), which was also confirmed by comparison of standardized partial regression coefficients from multiple repression (PW = 0.594 × GW + 0.841 × SLW–2.67E^-^17, *R*^2^ = 0.999, *P* < 0.0001, Fig 2B). No significant correlation was found between GW and SLW (Fig 2A, S2 Table).

We also performed principal component analysis to evaluate phenotypic integration among traits other than PW, GW, and SLW (Fig 2C). PC1 and PC2 accounted for 26.2% and 23.2% of the variation for RILs, respectively. PC1 was a vector for CL, PL, FLL, PN, and SN, whereas PC2 was a vector for HI, SF, DTH, and NSC.

### QTL mapping

**Fig 3:**
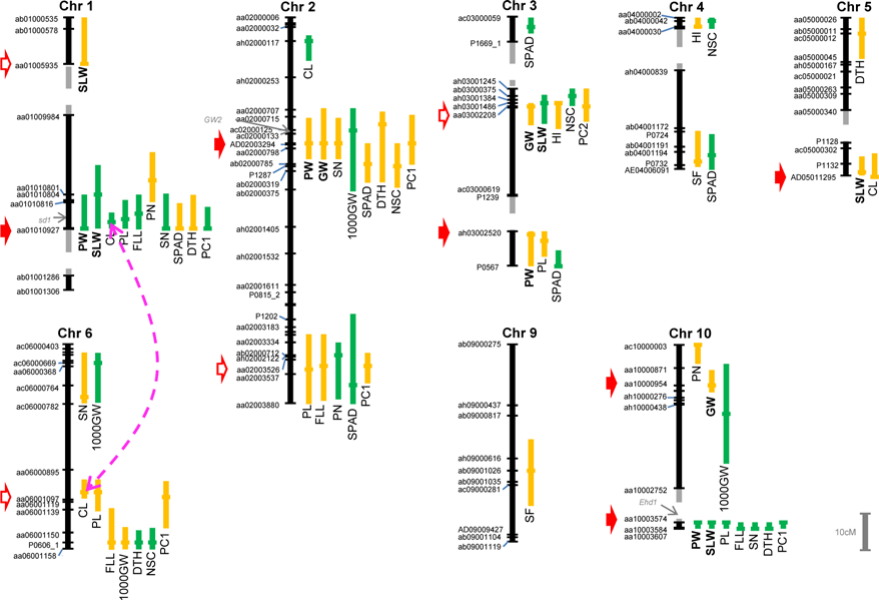
Locations of QTLs for biomass-related traits quantified in this study. Vertical bars to the right of the linkage map of each chromosome denote one-LOD confidence intervals; horizontal bars denote the position of the LOD peak at each QTL. Green signifies a QTL for which the ‘Tachisugata’ allele had a positive effect; yellow signifies a QTL for which the ‘Hokuriku 193’ allele had a positive effect. Gray broken lines how linkage gaps. Ten SNP markers subjected to multiple regression analysis are marked with red arrows; six of them (filled arrows) were chosen for QTL-based selection. A pink broken line connecting QTLs on Chrs 1 (aa01010927) and 6 (aa06001097) indicates significant epistasis (*p* = 0.0067). PW, plant weight; GW, grain weight; SLW, stem and leaf weight; HI, harvest index; CL, culm length; PL, panicle length; FLL, flag leaf length; PN, panicle number; SN, spikelet number per panicle; 1000GW, 1000-grain weight; SF, spikelet fertility; SPAD, chlorophyll content; DTH, days to heading; NSC, non-structural carbohydrate content. For visual clarity, Chrs 7, 8, 11, and 12, where no significant QTLs were detected, and the names of some markers are omitted.

Using composite interval mapping in RILs, we identified 55 QTLs for the 14 traits and 6 QTLs for PCs on chromosomes (Chrs) 1–6, 9, and 10 (Table 2, Fig 3). Among traits directly associated with biomass yield, four QTLs were mapped for PW (Chrs 1–3 and 10), three for GW (Chrs 2, 3, and 10), and five for SLW [Chrs 1 (2 QTLs), 3, 5, and 10]. Of these, two QTLs for PW and two QTLs for SLW, which were detected on Chrs 1 and 10, each explained more than 10% of the phenotypic variation for the corresponding trait.

**Table 2:** Summary of QTLs detected in the recombinant inbred lines from a cross between 'Tachisugata' and 'Hokuriku 193'.

Trait	Chromosome	Nearest	Position*^a^*	LOD	PVE*^b^*	Additive*^c^*
		marker	(Mb)	value	(%)	effect
PW	1	aa01010927	40.70	5.60	14.77	37.07
PW	2	AD02003294	10.00	3.61	6.17	-25.30
PW	3	ah03002520	35.09	3.03	5.48	-22.87
PW	10	aa10003574	22.30	5.41	10.67	31.01
GW	2	AD02003294	10.00	2.70	5.20	-13.15
GW	3	aa03002208	22.11	4.91	9.01	-16.29
GW	10	aa10000954	3.97	4.11	7.53	-15.09
SLW	1	aa01005935	11.42	2.99	5.48	-18.23
SLW	1	aa01010801	37.67	4.40	16.55	32.04
SLW	3	ah03001486	19.21	4.45	7.51	22.02
SLW	5	AD05011295	26.99	4.13	7.48	-21.23
SLW	10	aa10003574	22.30	5.99	12.04	27.56
HI	3	ah03001486	19.21	7.02	13.09	-2.07
HI	4	aa04000040	1.06	3.26	7.08	-1.51
CL	1	aa01010927	40.70	54.23	73.09	15.78
CL	2	ah02000117	2.63	3.75	2.78	3.06
CL	5	AD05011295	26.99	5.60	4.47	-3.71
CL	6	aa06001097	27.75	12.40	11.42	-5.94
PL	1	aa01010816	39.26	10.43	22.60	1.30
PL	2	aa02003526	31.39	3.61	6.49	-0.69
PL	3	ah03002520	35.09	4.17	7.33	-0.74
PL	6	aa06001097	27.75	8.50	15.43	-1.04
PL	10	aa10003574	22.30	2.80	4.91	0.60
FLL	1	aa01010816	39.26	17.41	44.36	2.67
FLL	2	aa02003537	31.98	5.81	10.45	-1.32
FLL	6	P0606_1	30.02	5.12	8.07	-1.15
FLL	10	aa10003584	22.58	2.66	3.74	0.78
PN	1	aa01010801	37.67	14.80	39.23	-0.66
PN	2	ab02000712	30.62	5.30	8.30	0.31
PN	10	ac10000003	0.04	2.59	3.67	-0.20
SN	1	aa01010927	40.70	4.48	9.38	11.35
SN	2	AD02003294	10.00	6.90	12.36	13.66
SN	6	ac06000764	22.58	3.49	13.27	-13.08
SN	10	P1771	22.47	2.61	4.86	8.21
1000GW	2	ac02000125	7.82	4.81	12.03	0.89
1000GW	6	ac06000669	20.66	5.67	11.72	0.84
1000GW	6	P0606_1	30.02	3.36	6.92	-0.65
1000GW	10	ah10000438	10.98	4.29	10.04	0.78
SF	4	P0732	28.77	6.54	13.61	-2.26
SF	9	ab09001026	16.20	2.56	5.46	-1.63
SPAD	1	aa01010927	40.70	4.90	9.37	-0.75
SPAD	2	aa02000798	10.15	5.57	10.72	-0.83
SPAD	2	aa02003537	31.98	3.06	7.93	0.67
SPAD	3	P1669_1	6.52	3.04	5.99	0.60
SPAD	3	P0567	37.23	3.27	6.21	0.60
SPAD	4	ab04001194	27.11	5.01	10.31	0.76
DTH	1	aa01010927	40.70	2.64	4.26	-0.60
DTH	2	aa02000715	7.82	4.80	10.45	-0.97
DTH	5	ab05000011	0.98	3.76	5.88	-0.69
DTH	6	P0606_1	30.02	2.65	4.19	0.57
DTH	10	aa10003607	22.58	11.52	19.03	1.25
NSC	2	P1287	14.54	2.76	5.06	-1.94
NSC	3	ab03000375	16.51	5.83	10.52	2.63
NSC	4	ab04000042	N/A	3.05	5.70	1.92
NSC	6	P0606_1	30.02	2.77	5.26	1.87
PC1	1	aa01010927	40.70	33.89	51.15	1.26
PC1	2	AD02003294	10.00	3.69	3.36	-0.34
PC1	2	aa02003526	31.39	11.15	12.15	-0.61
PC1	6	aa06001097	27.75	8.76	9.53	-0.53
PC1	10	aa10003574	22.30	3.10	3.11	0.31
PC2	3	aa03002208	22.11	3.09	6.02	-0.41

^*a*^ Position is based on the physical position within the rice genome (Build 4, http://rgp.dna.affrc.go.jp/) of the cultivar 'Nipponbare' (O. sativa ssp. japonica)

^*b*^ Phenotypic variance explained (R2 value x 100)

^*c*^ Additive effects in this column refer to the effect of the allele from 'Tachisugata' relative to that from 'Hokuriku 193'

PW, plant weight; GW, grain weight; SLW, stem and leaf weight; HI, harvest index; CL, culm length; PL, panicle length; FLL, flag leaf length; PN, panicle number; SN, spikelet number per panicle; 1000GW, 1000-grain weight; SF, spikelet fertility; SPAD, chlorophyll content; DTH, days to heading; NSC, non-structural carbohydrate content.

QTLs detected for the other traits were as follows: two QTLs for HI (Chrs 3 and 4); four for CL (Chrs 1, 2, 5, and 6); five for PL (Chrs 1–3, 6, and 10); four for FLL (Chrs 1, 2, 6, and 10); three for PN (Chrs 1, 2,and 10); four for SN (Chrs 1, 2, 6, and 10); four for 1000GW (Chrs 2, 6, and 10); two for SF (Chrs 4 and 9); seven for SPAD (Chrs 1–4); five for DTH (Chrs 1, 2, 5, 6, and 10); and four for NSC (Chrs 2–4 and 6). Five QTLs for PC1 (Chrs 1, 2, 6, and 10) and one for PC2 (Chr 3) were also mapped.

It should be noted that QTL(s) for PW, CL, PL, FLL, and PC1, which have a LOD peak near aa01010816 (at 39.3 Mb) or aa01010927 (at 40.7 Mb) on Chr 1, each accounted for 14.8–73.1% of the phenotypic variation for the corresponding trait (Table 2). Phenotypic variations of CL (73.1%), PL (22.6%), and FLL (44.4%) were particularly well explained by the QTL(s).

### QTL clusters

When one-LOD confidence intervals of three or more QTLs overlapped, we considered these QTLs to be a cluster. Seven QTL clusters were found in RILs on Chrs 1–3, 6, and 10, with two clusters on each Chr 2 and 3 (Fig 3). The presence of QTL clusters is supported by the observation that almost all clusters contained a QTL for a principal component (*i*.*e*., an integrated trait); the only exception was a cluster on Chr 3 (near ah03002520, at 35.1 Mb).

### Tests for QTL × QTL interactions

No significant epistatic interaction was detected between QTLs for the 13 traits, including PW, GW, and SLW, suggesting additive relationships between these QTLs. Only one pair of QTLs for CL, consisting of the QTL on Chr 1 (aa01010927) and the one on Chr 6 (aa06001097), showed a significant epistatic interaction (*P* = 0.0067, Fig 3).

### Candidate genes

Since QTL mapping was performed using only small numbers of RILs and markers (both <200), it was difficult to determine the relationships between detected QTLs and known genes. Still, the relationships between traits and QTL positions suggest some candidate genes. QTLs for CL, PL, and FLL (and possibly for PW, SN, SPAD, and DTH) in a cluster detected on Chr 1, in which the LOD peaks were detected between aa01010927 and aa01010816 (the nearest markers), may correspond to the *semi-dwarf 1* (*sd1*) locus at 40.1 Mb (Table 2, Fig 3) [3, 4]. Comparison of the sizes of PCR products followed by partial sequencing of the gene revealed that TS carries a functional allele, whereas H193 carries a nonfunctional allele with a critical deletion in the gene, consistently with the direction of the additive effects of the respective alleles (S2 Fig).

The QTL for 1000GW on Chr 2 may correspond to the *GW2* locus at 8.2 Mb [28] and that for DTH on Chr 10 to the *Early heading date 1* locus at 17.6 Mb [29]; Table 2, Fig 3).

### Combined effect of detected QTLs on PW

**Fig 4:**
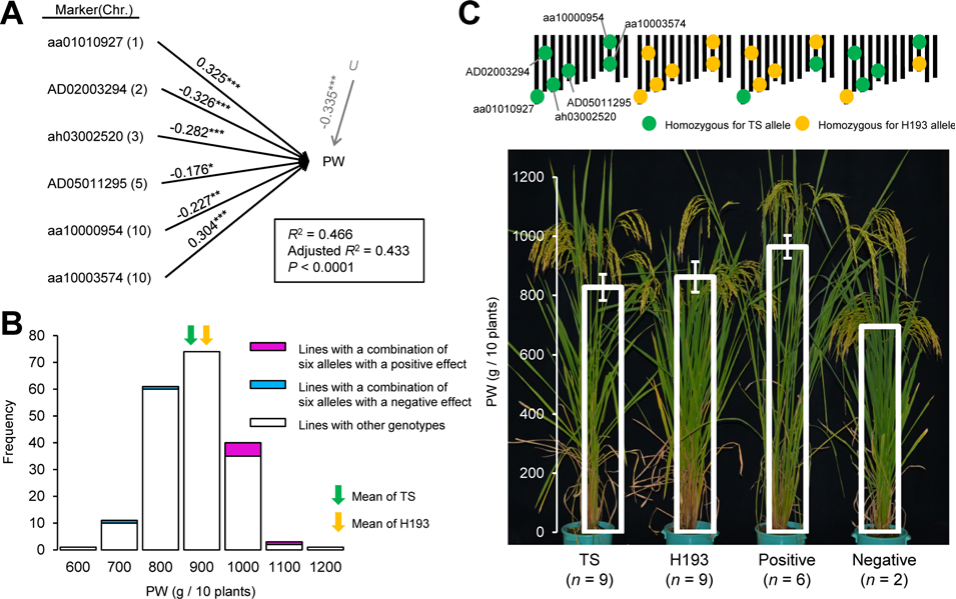
Combined effect of QTLs for plant weight in recombinant inbred lines. (A) Path diagram showing the nearest marker to each QTL and the residual variable *U*, affecting plant weight (PW). Nearest marker loci to QTLs for each trait were used as variables. Numbers in parentheses indicate chromosomal locations. Numbers on the lines indicate partial regression coefficients calculated by multiple regression analysis. *, 0.05 > *P* > 0.01; **, 0.01 > *P* > 0.001; ***, 0.001 > *P*. (B) Frequency distribution of PW. (C) Genotypes and PW of parental cultivars and selected lines. Means ±SD for each genotype class are shown. TS, ‘Tachisugata’; H193, ‘Hokuriku 193’.

To assess the combined effect of detected QTLs on PW in RILs, we performed multiple regression analysis. First, eight QTLs for PW (on Chrs 1–3), GW (on Chr 3 and 10), and SLW (two on Chrs 1 and one on Chr 5), and two QTLs for PC1 (on Chrs 2 and 6) were chosen. In total, 10 SNP markers nearest to the LOD peak of each QTL were analyzed (red arrows in Fig 3). Six of the markers, which were selected as variables in the best-fitting model chosen using the corrected Akaike information criterion (filled red arrows in Fig 3, Fig 4A), accounted for 43.3% of the phenotypic variation for PW (*P* < 0.0001). Indeed, lines selected from RILs on the basis of the allele types at the six QTLs were distributed according to their genotypes (Fig 4B); the mean PW values of the selected lines (1036.0 ± 91.9 g for alleles with a positive effect and 810.5 g for alleles with a negative effect) and their parents (852.2 ± 42.1 g for TS and 970.9 ± 154.3 g for H193) were also distinguishable from one another, as expected (Fig 4C).

### Evaluation of QTL-based selection for PW

**Fig 5:**
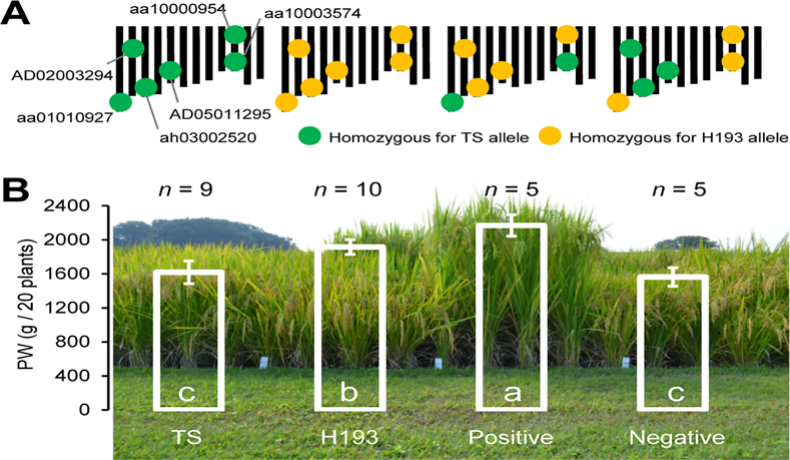
Effect of QTL-based selection on plant weight in the F_6_ progeny. (A) Genotypes of parental varieties and selected lines. (B) Plant weight (PW) of parental cultivars and selected lines. Means ±SD for each genotype class are shown. Means with different letters are significantly different (Tukey–Kramer HSD test, α = 0.05). TS, ‘Tachisugata’; H193, ‘Hokuriku 193’.

From F_2_ plants, we divergently selected lines that carried alleles with positive and negative additive effects at the six QTLs and compared PW between F_6_ progeny of the selected lines and their parents (S3 Fig, Fig 5). It should be noted that we did not select one of the QTLs (aa10000954) in the negatively selected lines (Fig 5).

Significant divergence was observed between the mean PW values of the positively (2169.0 ± 125.0 g) and negatively (1563.6 ± 108.2 g) selected lines; moreover, the mean value of positively selected lines was significantly higher than those of TS (1617.9 ± 133.3 g) and H193 (1916.1 ± 87.3 g) (Fig 5).

Divergent selection affected SLW, CL, PL, SN, and DTH in the same manner as it affected PW. However, it showed adverse effects on HI, PN, 1000GW, and SF, in that the values of negatively selected lines were higher than those of positively selected lines (S4 Fig). GW was not affected by divergent selection. Thus, the improvement of PW (Fig 5) appeared to result from the increase of SLW rather than that of GW (S4 Fig).

## Discussion

In this study, we showed that classical QTL analysis has a high ability to map phenotypes to genotypes, and that QTL-based selection is effective in increasing rice PW, even when two high-yielding cultivars are used to develop RILs.

We found significant phenotypic correlations between traits. Multiple traits were significantly correlated with one or more biomass traits (PW, GW, and SLW; Fig 2A, S2 Table). Our QTL analysis revealed the presence of QTL clusters; that is, colocalization of QTLs for multiple traits (Fig 3). QTL clusters are generally thought to result from pleiotropy of a single QTL or from tightly linked QTLs for multiple traits, either of which cases would result in genetic correlation [30, 31]. Thus, the phenotypic correlations we found may, at least in part, result from genetic correlations. The presence of a QTL for the target trait will be more reliable if multiple traits are analyzed; moreover, such QTLs can be reliable candidate markers for selection aimed at improving the target trait.

In contrast, because of genetic correlation, it seems likely that a tradeoff between traits may result in hidden genetic variation in PW. Both GW and SLW are component traits of PW, and HI and NSC play important roles in determining GW and SLW ([32] for HI; [22] for NSC). Our analysis of phenotypic variation showed positive correlations between GW and HI and between SLW and NSC, but negative correlations between GW and NSC, HI and SLW, and HI and NSC (Fig 2A, S2 Table). Such phenotypic relationships may be accounted for, in part, by the presence of QTL clusters. For example, in the QTL cluster on Chr 3 (aa03002208 as a representative marker), H193 alleles had a positive additive effect for the QTLs for GW and HI, but TS alleles had a positive additive effect for the QTLs for SLW and NSC (Table 2, Fig 3); thus, this QTL region may not account for PW variation in the multiple regression (Fig 4A). It should be noted that these results imply that GW and SLW are potentially interconnected through other traits such as HI and NSC, despite the absence of a significant correlation between GW and SLW in RILs, suggesting additive contributions of these traits to PW (Fig 2A).

By enabling measurements of bulks of individuals (or families) with replication, the use of biparental RILs was helpful in biomass yield phenotyping, which must detect QTLs with small effects. Thus, QTL-based selection will be dependable for improving rice biomass yield if biparental crosses are used.

In conventional rice breeding programs, crosses between a large number of varieties or candidate lines are conducted and the progeny are subjected to selection. Considering that multiple alleles are introduced and that breeders are interested in alleles associated with desirable phenotypes in diverse varieties, genome-wide association studies (GWAS) may be more suitable for QTL mapping than QTL analysis. GWAS have succeeded in detecting QTLs for agriculturally important traits in rice [16, 33, 34]. However, one should keep in mind that GWAS have some weak points such as the detection of false-positive or negative QTLs in the presence of population structure and low power to detect rare alleles in mapping populations because the detection method depends on allele frequency [35, 36]. The development of well-designed mapping populations known as nested association mapping (NAM) populations and multi-parent advanced generation intercross (MAGIC) populations, which segregate for multiple alleles, is expected to solve these problems [37, 38]. As with GWAS, haplotype analysis based on pedigree, which employs mapping populations with multiple alleles, can help us to find haplotype blocks or genomic regions selected by breeders [15, 20].

It has been recognized that because there is no one-size-fits-all approach in QTL mapping that is applicable to all mapping populations, the most suitable approach should be applied in each study [39]. If possible, the QTL effect should be validated by a combination of different approaches; for example, biparental QTL analysis after GWAS or haplotype analysis will provide additional information useful for the improvement of the target trait [39].

Another approach for genomics-assisted selection, genomic selection (GS), is based on genomic breeding values. Initially proposed for livestock breeding, GS has been recently extensively evaluated for crop improvement [40, 41]. Genomic breeding values are calculated as the sum of the effects of genetic markers across the entire genome of individuals in a reference (training) population; this approach potentially captures all QTLs that contribute to phenotypic variation in a trait, even if they have minor effects. More recently, GS has yielded promising results in rice, although the phenotypic performance of selected lines has not yet been validated [42–44].

Finally, the connection between genotypes and phenotypes is the most important key in all genomics-assisted selection approaches. Whereas current advances in rice genomic research have facilitated genotyping at the whole-genome level, phenotyping of complex traits, such as biomass yield will remain a bottleneck, because it is laborious and field management is difficult. As Poland [45] has pointed out, in field-based research, collaboration between genomics and conventional breeding programs, which always excel in phenotyping of multiple traits (such as biotic and abiotic stress tolerance and grain quality) and in multiple environments (locations and years), is essential to further accelerate genotype-to-phenotype mapping of agriculturally important traits such as biomass yield.

## Supporting Information

S1 Fig Frequency distributions of the values of 14 traits in recombinant inbred lines. Values along the x axis correspond to the trait and units given in the corresponding graph label. PW, plant weight; GW, grain weight; SLW, stem and leaf weight; HI, harvest index; CL, culm length; PL, panicle length; FLL, flag leaf length; PN, panicle number; SN, spikelet number per panicle; 1000GW, 1000-grain weight; SF, spikelet fertility; SPAD, chlorophyll content; DTH, days to heading; NSC, non-structural carbohydrate content. Distribution normality was examined by Shapiro–Wilk test. * shows a departure from normality. TS, ‘Tachisugata’; H193, ‘Hokuriku 193’.
(PPTX)

S2 Fig Comparison of *semi-dwarf 1* alleles carried by ‘Tachisugata’ and ‘Hokuriku 193’. (A) Structure of the *sd1* gene and position of PCR primers used in this experiment. (B) Electrophoretic profile of PCR products. PCR analyses were performed using two pairs of primers: sd1_1F (CAGACAGCTCGCCCTGCA) and sd1_1R (CTGTTGCTTCGAAGCAGAGG) (the present study), and sd1-del-1U (ACGGGTTCTTCCAGGTGTC) and sd1-del-1L (CTGCTGTCCGCGAAGAACTC) [4]. M, marker. (C) Schematic representation of a deletion in the ‘Hokuriku 193’ allele. Sequence analyses were performed by direct sequencing of the products of nested PCR (PCR products that were amplified using a pair of sd1_1F/sd1_1R primers were used as templates for PCR using a pair of sd1_del_1U/sd1_del_1R primers).
(PPTX)

S3 Fig Genotypes of F_2_ plants selected on the basis of QTL analysis. (A) A line that carried alleles with positive additive effects at six QTLs. (B) A line that carried alleles with negative additive effects at six QTLs. Green and orange horizontal bars show ‘Tachisugata’ and ‘Hokuriku 193’ alleles at the marker loci, respectively. Gray horizontal bars represent heterozygous at the marker loci. Positions of the markers nearest to the selected QTLs are shown by red triangles.
(PPTX)

S4 Fig Effect of QTL-based selection on 10 traits in the F_6_ progeny. PW, plant weight; GW, grain weight; SLW, stem and leaf weight; HI, harvest index; CL, culm length; PL, panicle length; FLL, flag leaf length; PN, panicle number; SN, spikelet number per panicle; 1000GW, 1000-grain weight; SF, spikelet fertility; SPAD, chlorophyll content; DTH, days to heading; NSC, non-structural carbohydrate content. Means ± SD of each genotype class are shown. *n* = 9 for ‘Tachisugata’ (TS), *n* = 10 for ‘Hokuriku 193’ (H193), and *n* = 5 for the positive and negative selections. Means with different letters are significantly different (Tukey–Kramer HSD test).
(PPTX)

S1 File Genotypic and Phenotypic data for QTL mapping in the RILs. (XLSX)

S1 Table Primers used for genotyping of SNP markers. (XLSX)

S2 Table The Pearson’s correlation coefficients (above the diagonal) between traits and their corresponding *P* values (below the diagonal). (XLSX)
