# Supplementary material for: Improvement of Rice Biomass Yield through QTL-Based Selection
Source: PLoS One. 2016 Mar 17;11(3):e0151830. doi: 10.1371/journal.pone.0151830 (PMC4795639; doi:10.1371/journal.pone.0151830)
Supplement: S1 Fig — Values along the x axis correspond to the trait and units given in the corresponding graph label. PW, plant weight; GW, grain weight; SLW, stem and leaf weight; HI, harvest index; CL, culm length; PL, panicle length; FLL, flag leaf length; PN, panicle number; SN, spikelet number per panicle; 1000GW, 1000-grain weight; SF, spikelet fertility; SPAD, chlorophyll content; DTH, days to heading; NSC, non-structural carbohydrate content. Distribution normality was examined by Shapiro–Wilk test. * shows a departure from normality. TS, ‘Tachisugata’; H193, ‘Hokuriku 193’. (PPTX) [file pone.0151830.s001.pptx]

## Slide 1
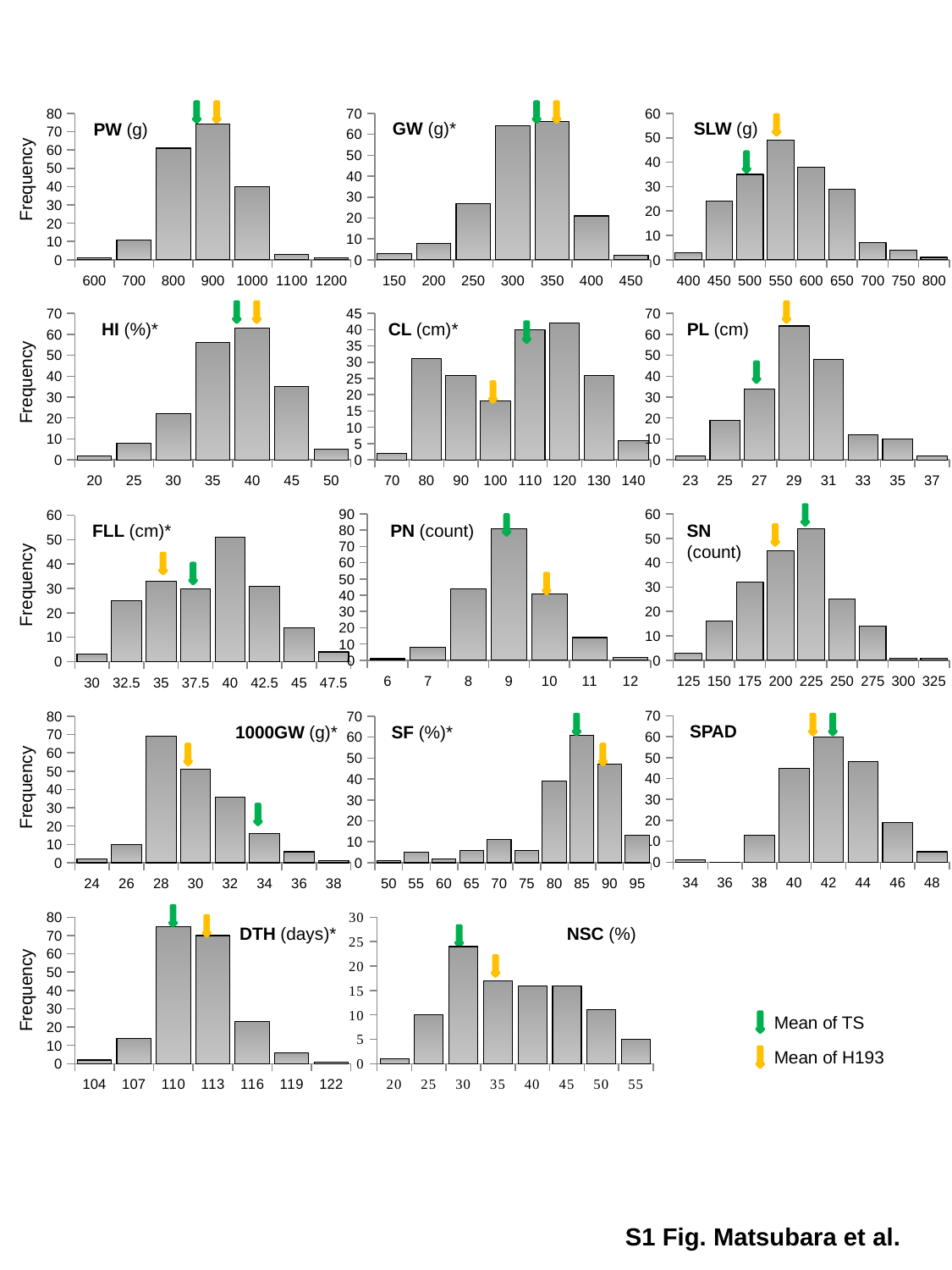

### Chart
| Category | |
|---|---|
| 600 | 1.0 |
| 700 | 11.0 |
| 800 | 61.0 |
| 900 | 74.0 |
| 1000 | 40.0 |
| 1100 | 3.0 |
| 1200 | 1.0 |PW (g)
### Chart
| Category | |
|---|---|
| 150 | 3.0 |
| 200 | 8.0 |
| 250 | 27.0 |
| 300 | 64.0 |
| 350 | 66.0 |
| 400 | 21.0 |
| 450 | 2.0 |GW (g)*
### Chart
| Category | |
|---|---|
| 400 | 3.0 |
| 450 | 24.0 |
| 500 | 35.0 |
| 550 | 49.0 |
| 600 | 38.0 |
| 650 | 29.0 |
| 700 | 7.0 |
| 750 | 4.0 |
| 800 | 1.0 |
SLW (g)
Frequency
### Chart
| Category | |
|---|---|
| 20 | 2.0 |
| 25 | 8.0 |
| 30 | 22.0 |
| 35 | 56.0 |
| 40 | 63.0 |
| 45 | 35.0 |
| 50 | 5.0 |HI (%)*
### Chart
| Category | |
|---|---|
| 70 | 2.0 |
| 80 | 31.0 |
| 90 | 26.0 |
| 100 | 18.0 |
| 110 | 40.0 |
| 120 | 42.0 |
| 130 | 26.0 |
| 140 | 6.0 |CL (cm)*
### Chart
| Category | |
|---|---|
| 23 | 2.0 |
| 25 | 19.0 |
| 27 | 34.0 |
| 29 | 64.0 |
| 31 | 48.0 |
| 33 | 12.0 |
| 35 | 10.0 |
| 37 | 2.0 |PL (cm)
Frequency
### Chart
| Category | |
|---|---|
| 6 | 1.0 |
| 7 | 8.0 |
| 8 | 44.0 |
| 9 | 81.0 |
| 10 | 41.0 |
| 11 | 14.0 |
| 12 | 2.0 |
### Chart
| Category | |
|---|---|
| 125 | 3.0 |
| 150 | 16.0 |
| 175 | 32.0 |
| 200 | 45.0 |
| 225 | 54.0 |
| 250 | 25.0 |
| 275 | 14.0 |
| 300 | 1.0 |
| 325 | 1.0 |SN
(count)
### Chart
| Category | |
|---|---|
| 30 | 3.0 |
| 32.5 | 25.0 |
| 35 | 33.0 |
| 37.5 | 30.0 |
| 40 | 51.0 |
| 42.5 | 31.0 |
| 45 | 14.0 |
| 47.5 | 4.0 |FLL (cm)*
PN (count)
Frequency
### Chart
| Category | |
|---|---|
| 34 | 1.0 |
| 36 | 0.0 |
| 38 | 13.0 |
| 40 | 45.0 |
| 42 | 60.0 |
| 44 | 48.0 |
| 46 | 19.0 |
| 48 | 5.0 |SPAD
### Chart
| Category | |
|---|---|
| 24 | 2.0 |
| 26 | 10.0 |
| 28 | 69.0 |
| 30 | 51.0 |
| 32 | 36.0 |
| 34 | 16.0 |
| 36 | 6.0 |
| 38 | 1.0 |1000GW (g)*
### Chart
| Category | |
|---|---|
| 50 | 1.0 |
| 55 | 5.0 |
| 60 | 2.0 |
| 65 | 6.0 |
| 70 | 11.0 |
| 75 | 6.0 |
| 80 | 39.0 |
| 85 | 61.0 |
| 90 | 47.0 |
| 95 | 13.0 |SF (%)*
Frequency
### Chart
| Category | |
|---|---|
| 104 | 2.0 |
| 107 | 14.0 |
| 110 | 75.0 |
| 113 | 70.0 |
| 116 | 23.0 |
| 119 | 6.0 |
| 122 | 1.0 |
DTH (days)*
### Chart
| Category | |
|---|---|
| 20 | 1.0 |
| 25 | 10.0 |
| 30 | 24.0 |
| 35 | 17.0 |
| 40 | 16.0 |
| 45 | 16.0 |
| 50 | 11.0 |
| 55 | 5.0 |NSC (%)
Frequency
Mean of TS
Mean of H193
S1 Fig. Matsubara et al.
