# Supplementary material for: Improvement of Rice Biomass Yield through QTL-Based Selection
Source: PLoS One. 2016 Mar 17;11(3):e0151830. doi: 10.1371/journal.pone.0151830 (PMC4795639; doi:10.1371/journal.pone.0151830)
Supplement: S2 Fig — (A) Structure of the sd1 gene and position of PCR primers used in this experiment. (B) Electrophoretic profile of PCR products. PCR analyses were performed using two pairs of primers: sd1_1F (CAGACAGCTCGCCCTGCA) and sd1_1R (CTGTTGCTTCGAAGCAGAGG) (the present study), and sd1-del-1U (ACGGGTTCTTCCAGGTGTC) and sd1-del-1L (CTGCTGTCCGCGAAGAACTC) [4]. M, marker. (C) Schematic representation of a deletion in the ‘Hokuriku 193’ allele. Sequence analyses were performed by direct sequencing of the products of nested PCR (PCR products that were amplified using a pair of sd1_1F/sd1_1R primers were used as templates for PCR using a pair of sd1_del_1U/sd1_del_1R primers). (PPTX) [file pone.0151830.s002.pptx]

## Slide 1
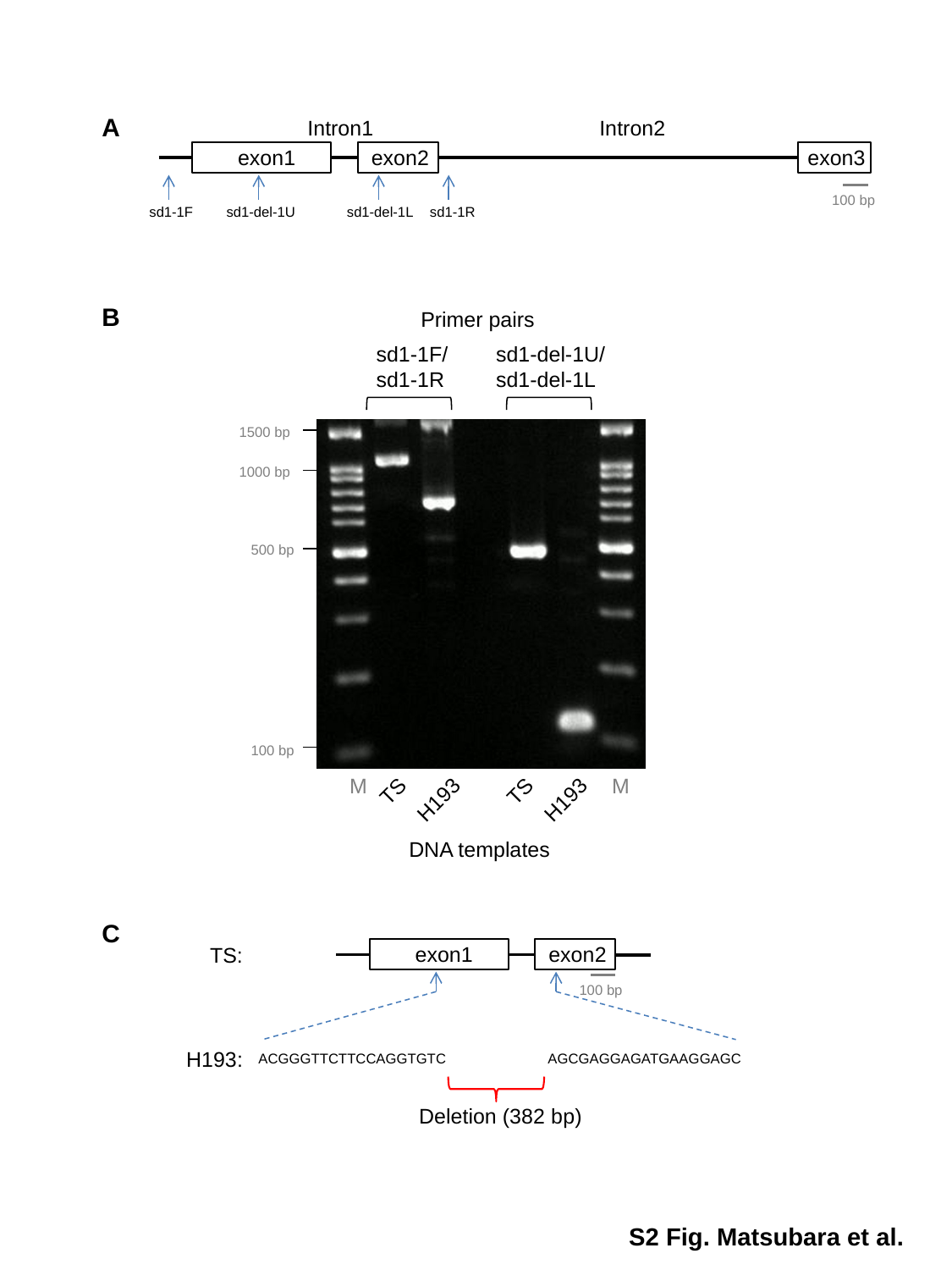

A
Intron1
Intron2
exon1
exon2
exon3
100 bp
sd1-1F
sd1-del-1U
sd1-del-1L
sd1-1R
B
Primer pairs
sd1-1F/
sd1-1R
sd1-del-1U/
sd1-del-1L
1500 bp
1000 bp
500 bp
100 bp
M
M
TS
TS
H193
H193
DNA templates
C
exon1
exon2
TS:
100 bp
H193:
ACGGGTTCTTCCAGGTGTC
AGCGAGGAGATGAAGGAGC
Deletion (382 bp)
S2 Fig. Matsubara et al.
