# Supplementary figures and images for: Improvement of Rice Biomass Yield through QTL-Based Selection
Source: PLoS One. 2016 Mar 17;11(3):e0151830. doi: 10.1371/journal.pone.0151830 (PMC4795639; doi:10.1371/journal.pone.0151830)

## Slide 1
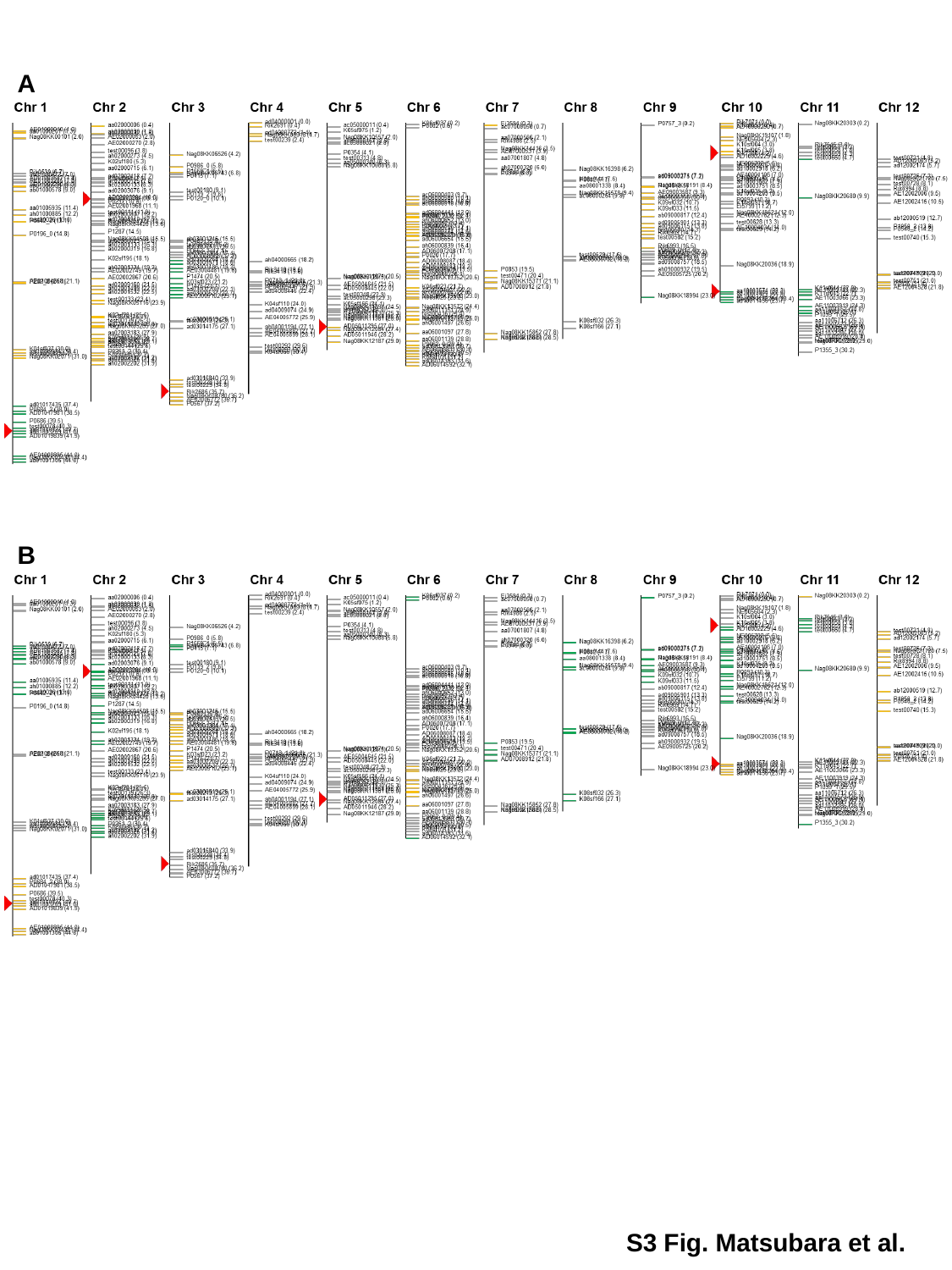

A
B
S3 Fig. Matsubara et al.

Supplement: S3 Fig — (A) A line that carried alleles with positive additive effects at six QTLs. (B) A line that carried alleles with negative additive effects at six QTLs. Green and orange horizontal bars show ‘Tachisugata’ and ‘Hokuriku 193’ alleles at the marker loci, respectively. Gray horizontal bars represent heterozygous at the marker loci. Positions of the markers nearest to the selected QTLs are shown by red triangles. (PPTX) [file pone.0151830.s003.pptx]
