# Supplementary material for: Improvement of Rice Biomass Yield through QTL-Based Selection
Source: PLoS One. 2016 Mar 17;11(3):e0151830. doi: 10.1371/journal.pone.0151830 (PMC4795639; doi:10.1371/journal.pone.0151830)
Supplement: S4 Fig — PW, plant weight; GW, grain weight; SLW, stem and leaf weight; HI, harvest index; CL, culm length; PL, panicle length; FLL, flag leaf length; PN, panicle number; SN, spikelet number per panicle; 1000GW, 1000-grain weight; SF, spikelet fertility; SPAD, chlorophyll content; DTH, days to heading; NSC, non-structural carbohydrate content. Means ± SD of each genotype class are shown. n = 9 for ‘Tachisugata’ (TS), n = 10 for ‘Hokuriku 193’ (H193), and n = 5 for the positive and negative selections. Means with different letters are significantly different (Tukey–Kramer HSD test). (PPTX) [file pone.0151830.s004.pptx]

## Slide 1
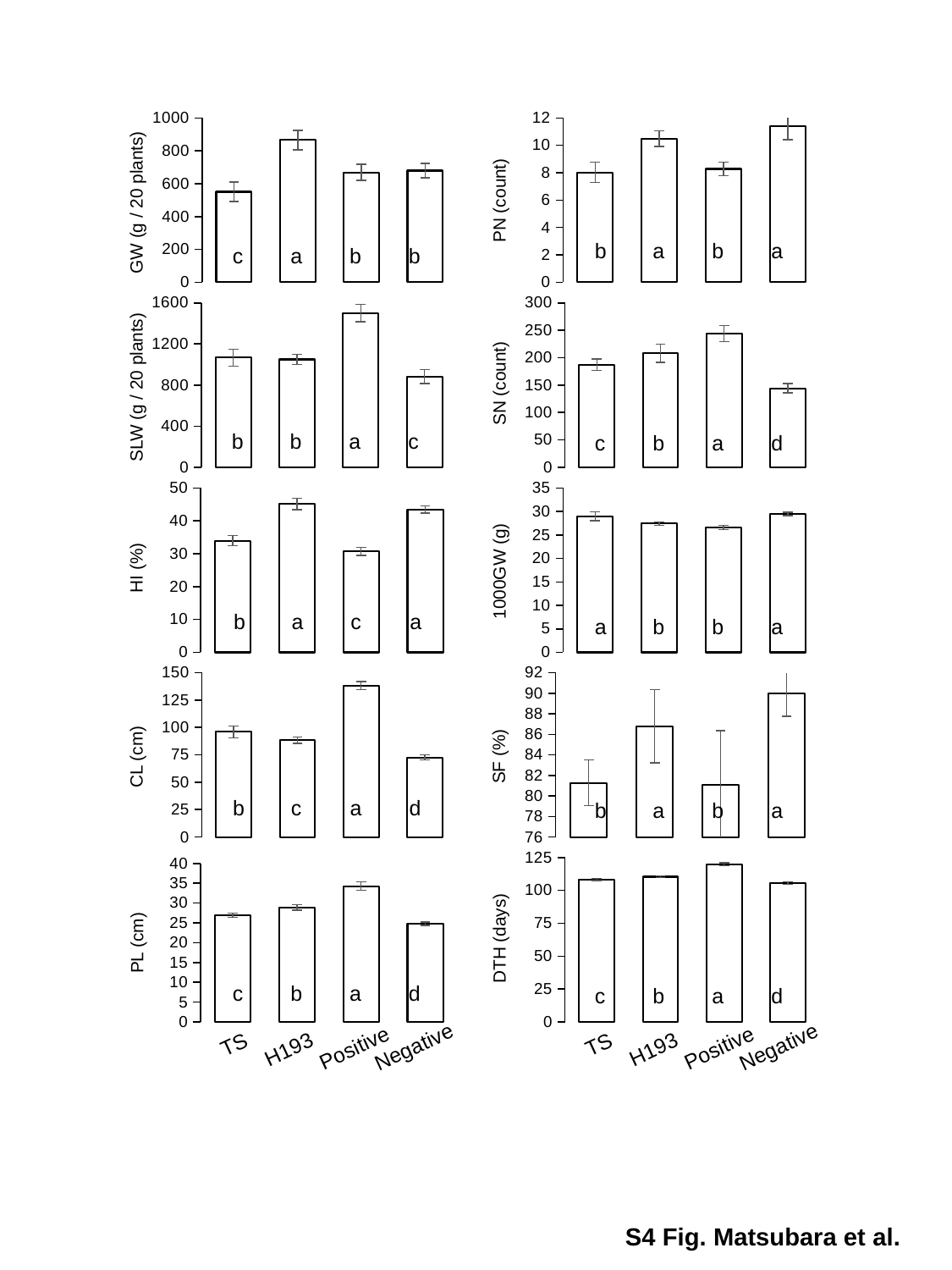

### Chart
| Category | |
|---|---|GW (g / 20 plants)
c
a
b
b
### Chart
| Category | |
|---|---|PN (count)
b
a
b
a
### Chart
| Category | |
|---|---|SLW (g / 20 plants)
b
b
a
c
### Chart
| Category | |
|---|---|SN (count)
c
b
a
d
### Chart
| Category | |
|---|---|HI (%)
b
a
c
a
### Chart
| Category | |
|---|---|1000GW (g)
a
b
b
a
### Chart
| Category | |
|---|---|CL (cm)
b
c
a
d
### Chart
| Category | |
|---|---|SF (%)
b
a
b
a
### Chart
| Category | |
|---|---|DTH (days)
c
b
a
d
### Chart
| Category | |
|---|---|PL (cm)
c
b
a
d
TS
TS
Negative
Negative
Positive
Positive
H193
H193
S4 Fig. Matsubara et al.
